# New Appliances and Things Medical

**Published:** 1902-03-01

**Authors:** 


### NEW APPLIANCES AND THINGS MEDICAL.

### HOVIS FOOD, Nos. 1 AND 2, FOR INFANTS AND INVALIDS.

(HOVIS BREAD FLOUR COMPANY, LIMITED, MACCLESFIELD.)

WE have received samples of the above food for examination. No. 1 food is an excellent example of a dextrinated soluble food, prepared from cereals, and containing a rich percentage of phosphates. Starch and cane sugar are absent from this preparation, and the modicum of cellulose which remains, is presented in a finely divided condition which is not likely to cause any irritation to the most delicate mucous membrane; in fact, its presence is calculated to afford a useful stimulus to the peristaltic movements of the bowel—when prepared according to the directions with diluted milk. The large excess of soluble carbohydrates is stated by the manufacturers to make good the deficiency of fat which occurs in such a mixture, but the substitution of an isodynamic quantity of carbohydrate for an equivalent proportion of fat, though theoretically sound, cannot be carried out successfully in practice. As a malted food this preparation is excellent; its reputation, however, will only suffer by claiming for it properties which cannot be substantiated. Moreover, we find considerable fault with the directions which are printed on the label. When the infant is a week old we are recommended to supply a tablespoonful of the food with each bottle of milk, that is to say, about ¼ lb. in the 24 hours, a quantity which, roughly stated, is nearly four times too much for safety. Food No. 2 is for infants of eight months old and upwards. It is only a partially dextrinated food and contains a small quantity of unaltered starch. It appears to be a very pure and carefully manufactured food and subject to the same criticism which we have made with respect to food No. 1, we regard it as a most desirable addition to milk which is intended for the nourishment of infants from the time that they begin to be able to digest amylaceous foods. For invalids both these foods are to be commended.

### ROWNTREE'S "ELECT" COCOA AND MILK CHOCOLATE.

(ROWNTREE AND CO., YORK, ENGLAND.)

IT is hardly necessary to reiterate the well-known properties of Rowntree's cocoa; this brand has long established for itself a reputation for excellence which is perhaps surpassed by no other cocoa in the market. A feature which will recommend itself to the economical is its high degree of concentration, while connoisseurs will approve its deliciously aromatic flavour. Chemical analysis has proved over and over again that it is impossible to impeach its purity. Rowntree's milk or cream chocolate is a comparatively new departure in the way of manufacture; it is a vast improvement on the original varieties of cake chocolate, and, whether for eating or cooking purposes, the enhanced nutritive value will be appreciated by its many patrons.

### CHURNO.

(VAN DEN BERGHS, LIMITED, 21 MINCING LANE, LONDON, E.C.)

CHURNO is a new variety of margarine which possesses qualities which will recommend it to the careful housewife. It is a preparation which is probably, in a chemical sense, much purer than most dairy butters, consisting as it does of neutral fats of almost identical composition with those which occur in fresh cream. From a nutritive standpoint there is little to be said in favour of either butter or margarine of the quality of Churno, nor as regards flavour has dairy butter any very material advantages, while as regards keeping properties and price the advantage is all on the side of Churno. The price is 8d. per lb., carefully packed in cardboard boxes.

